# Aberrant expression of FBXO22 is associated with propofol-induced synaptic plasticity and cognitive dysfunction in adult mice

**DOI:** 10.3389/fnagi.2022.1028148

**Published:** 2022-11-08

**Authors:** Xiaoxuan Yang, Chen Chen, Dongmei Qu, Yanping Liu, Ning Wang, Haibi Wang, Youjia Fan, Yushan Zhou, Buwei Yu, Qingsheng Xue, Yuqing Wu, Han Lu

**Affiliations:** ^1^Department of Anesthesiology, Ruijin Hospital, Shanghai Jiao Tong University School of Medicine, Shanghai, China; ^2^Jiangsu Province Key Laboratory of Anesthesiology, Xuzhou Medical University, Xuzhou, China; ^3^NMPA Key Laboratory for Research and Evaluation of Narcotic and Psychotropic Drugs, Xuzhou Medical University, Xuzhou, China

**Keywords:** propofol, FBXO22, hippocampus, general anesthesia, neurocognition

## Abstract

Recent observation demonstrated that prolonged anesthesia modifies brain synaptic architecture in all ages, including adult. Propofol is the most commonly utilized anesthetics at clinic. Whether repeated administration of propofol modulates cognitive impairment in adults and changes synaptic plasticity remains, however, to be explored. In this study, we first discovered that repeated and prolonged exposure to propofol-induced cognitive impairment in adult rodents. Then, we examined the property of hippocampal primary neurons and slices after propofol treatment in mice, including synaptic protein profile, dendritic spine density, as well as synaptic transmission. We found the distinctive change of the F-box only protein 22 (FBXO22), an F-box E3 ligase, during this process and further explored its role. Knockdown experiments showed the downregulation of FBXO22 restored the changes by propofol treatment on hippocampal primary neurons and attenuated propofol-induced hippocampal dependent cognitive dysfunction. Our results showed that FBXO22 is involved in the regulation of repeated propofol treatment induced changes of synaptic plasticity and cognitive dysfunction in adult mice. Repeated propofol treatment leads to cognitive dysfunction by regulating FBXO22 in adult rodents.

## Key points

Question: The underlying mechanism of propofol-induced amnesia is still unknown.

Findings: propofol modulates memory acquisition *via* an activation of the F-box only protein 22 (FBXO22), an F-box ubiquitin-conjugating ligase.

Meaning: FBXO22 is a potential target for treating propofol-induced cognitive dysfunction.

## Introduction

Propofol is a short-acting intravenous general anesthetic most commonly utilized in the induction and maintenance of general anesthesia as well as in invasive examination and sedation in intensive care units (Wheeler et al., 2003). The influence of general anesthesia on cognitive impairment is controversial and complex. In early life, when the brain is highly plastic, repeated general anesthesia has been shown to result in decreased synaptic density and long-term cognitive impairment (De Roo et al., 2009; Briner et al., 2011). The general view at present is that during later adolescence and adulthood, dendrites and dendritic spines are stable under physiological conditions (Grutzendler et al., 2002; Zuo et al., 2005) and short-term exposure to general anesthesia (Yang et al., 2011). However, recent study proved that prolonged general anesthesia (e.g., isoflurane) changed synaptic architecture and object recognition at all ages (Wenzel et al., 2021). This notion suggests that the synaptic structure of the adult brain is not as stable as previously thought for long-term administration of general anesthesia. Therefore, it is critical to determine the safest doses of anesthesia for adult patients who need repeated general anesthesia at clinics. In addition, it is necessary to evaluate cognitive function after repeated administration of propofol during later adolescence and adulthood and study the changes of synaptic plasticity.

There are currently no standard methods to prevent cognitive dysfunction caused by anesthesia, mostly because the mechanism of this process is not completely understood. Previous studies mainly focused on the effects of general anesthetics on key proteins regarding the formation of fear memory (Liu et al., 2014). However, it is well-known that protein levels depend on the dynamic balance between protein synthesis and degradation. In the past decade, studies of an increasing number have revealed that the ubiquitin proteasome system (UPS) is pivotal in the formation of memory (Hegde, 2017; Jarome and Devulapalli, 2018). There has been plentiful evidence implying that ubiquitin-proteasome-induced degradation has a role in the molecular mechanisms of synaptic plasticity that underly memory (Hegde, 2004; Hegde et al., 2014). Our previous work showed that sevoflurane acts on the ubiquitination-proteasome pathway to facilitate the degradation of post-synaptic density 95 (PSD95) protein (Lu et al., 2017), although it is not yet known whether the adverse effects of propofol on cognitive function involve regulation of the UPS.

The SKP1-Cullin-F-box protein (SCF) E3 ligase complex is the largest family of E3 ubiquitin ligases in eukaryotes, regulating the ubiquitin-dependent proteolysis of key regulatory proteins (Ciechanover, 2015). The F-box protein component binds to SKP1 and Cullin 1 *via* the F-box domain and binds to the substrates *via* other motifs involving interactions between proteins (Skaar et al., 2013; Wang et al., 2014), so as to determine the substrate specificity. FBXO22 (F-box only protein 22) is a novel, well-characterized F-box protein (Tan et al., 2011; Tian et al., 2015; Johmura et al., 2016). Significant efforts have been devoted to identify the emerging critical role of FBXO22 in cancer (Zhang et al., 2019; Zhu et al., 2019). However, studies on FBXO22 and the central nervous system are still scarce. One study showed that FBXO22 has a key role in regulating D-Serine synthesis (Elena et al., 2014), which mediates numerous NMDAR-mediated processes ranging from normal neurotransmission to neurodegeneration. GSE35642 database revealed that FBOX22 is upregulated in an animal model of Parkinson’s disease (PD) *via* the mechanism of promoting the ubiquitination-dependent degradation of PHLPP1 to ameliorate neurotoxicity, revealing the role of FBXO22 in PD occurrence and progression (Zheng et al., 2021). The above evidence indicated that FBXO22 may be associated with neurological diseases. Therefore, we intend to further explore the role of FBXO22 during the process of repeated propofol anesthesia on synapses plasticity and cognitive function.

The current study tests the hypothesis that repeated propofol treatment impairs cognitive function by increasing protein levels of FBXO22 in the hippocampus. We demonstrate that the down-regulation of FBXO22 by specific shRNA restores the decreased glutamatergic synaptic transmission and spine density and attenuates the long-term learning deficits induced by propofol, thus better characterizing the effects of repeated anesthesia on synapses plasticity and cognitive function, which provides further basic evidence for a more individualized anesthesia scheme for clinics (Brown et al., 2018).

## Materials and methods

### Experimental animals and drug treatment

The study was pre-registered and approved by the Laboratory Animal Ethics Committee of Xuzhou Medical University (L20210113006). Male C57BL/6 mice weighing 23–30 g were used. Housed in standard rodent cages, the mice were fed for at least 2 weeks under a 12-h light/dark cycle (lights on at 6:00 am), with free access to food and water before the start of the experiments. All behavioral experiments were conducted between 10:00 and 17:00.

**Figure 1:**
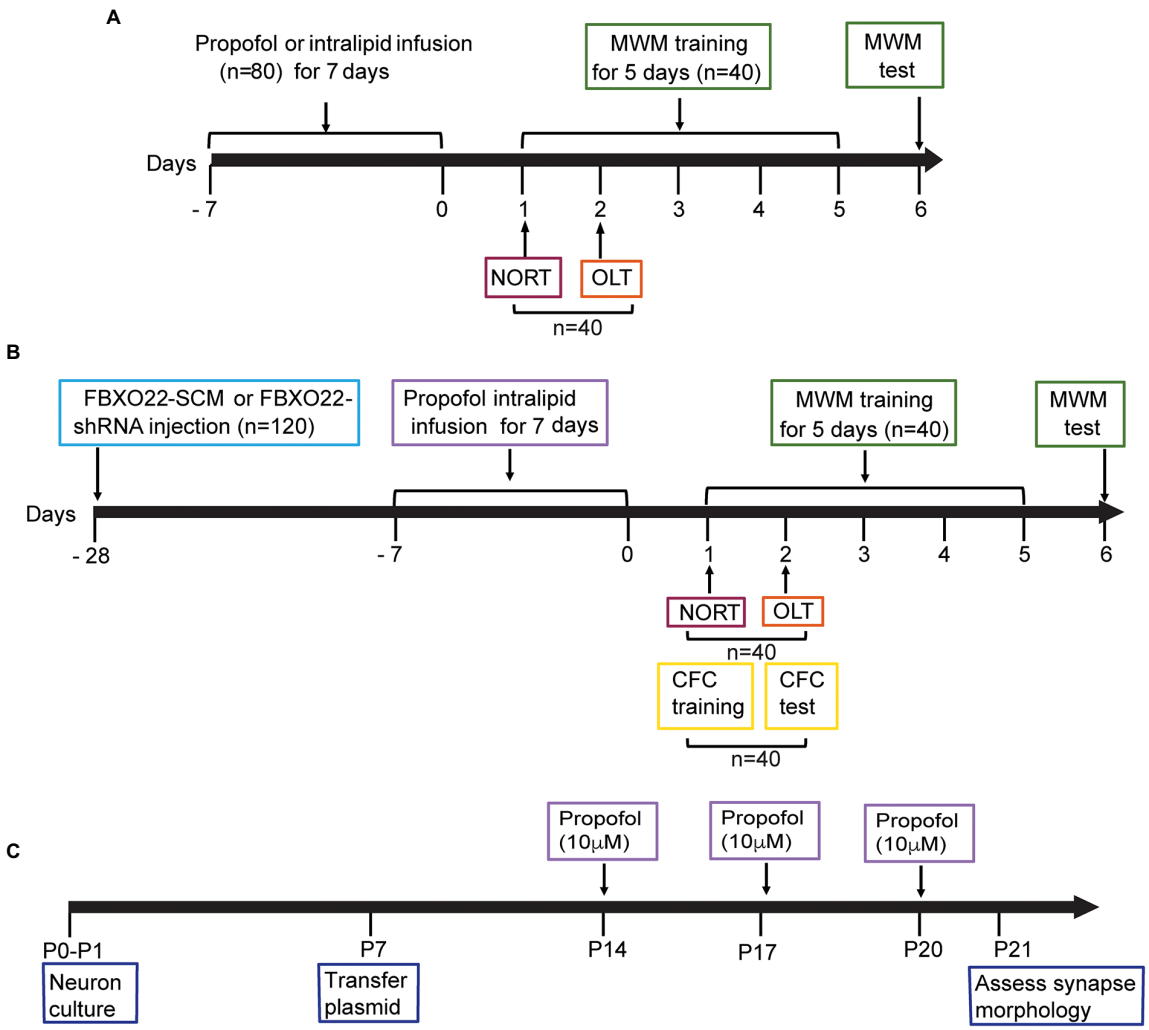
Diagram of timeline of experimental procedures. **(A)** Effects of different doses of propofol anesthesia on spatial learning and memory in mice. 80 mice were randomly allocated to two behavioral experiments: Morris water maze (MWM): 4 groups (control, propofol 25 mg/kg, propofol 50 mg/kg, propofol 100 mg/kg) × 10 mice/group. The novel object recognition test (NORT) and object location test (OLT): 4 groups (the same group allocation as MWM) × 10 mice/group. **(B)** The effect of FBXO22 regulation on cognitive dysfunction induced by propofol. 120 mice were randomly allocated to three behavioral experiments. MWM: 4 groups (control+FBXO22-SCM, control+FBXO22-shRNA, propofol+FBXO22-SCM, propofol+ FBXO22-shRNA) × 10 mice/group; NORT and OLT: 4 groups (the same group allocation as morris water maze) × 10 mice/group; Contextual fear conditioning (CFC), the same group allocation as MWM: 4 groups×10 mice/group. **(C)** The schematic time course and experimental designs of spine density model P: postnatal.

In preliminary experiments, the dose of propofol (AstraZeneca, UK Limited) at which the righting reflex was abolished was 50 mg/kg. The dose of propofol which resulted in the loss of the righting reflex in >95% of adult mice was 200 mg/kg (Irifune et al., 1999). So we chose propofol dosage from subanesthetic to high, mice were administered a single dose of propofol (10, 25, 50, 100, and 200 mg/kg, i.p.) for consecutive 7 days. Control animals received Intralipid vehicle injections (Sigma, St. Louis, MO, United States). The detail of animals’ treatment is shown in Figure 1.

### Behavioral experiments

There were 4 groups for behavioral experiments, control group and three dosage (25, 50, and 100 mg/kg) of propofol (*N* = 10 for each group), or 100 mg/kg once a day for 7 days in the FBXO22-shRNA rescue experiment, in an additional cohort of animals. We set such a grouping to achieve our goal, reduce the animal amount and avoid potential waste. The schematic diagram for the behavioral experiment is shown in Figure 1.

The morris water maze (MWM) test measures hippocampal-dependent spatial learning and memory ability of the mice, and was conducted according to our previous study (Lu et al., 2017). The experimental apparatus consisted of a circular white background pool (diameter 120 cm, height 30 cm) with clean water (22–24°C) and a movable transparent escape platform (diameter 10 cm, height 24 cm). The perimeter of the pool was completely surrounded from ceiling to floor by blue curtains. The pool was divided into four quadrants, each marked with four white cardboard shapes (square, triangle, circle, and cross) on the wall of the pool as spatial clues. The swimming pool was filled with water to a depth of 25 cm, while the unseen platform was immersed 1 cm below the surface of the water and placed in the center of quadrant IV. The four starting points were equidistant from the pool edges of quadrants I, II, III, and IV. Testing in a dark environment, mice were given 60 s to find the platform, and whether they found it or not, they were forced to stay on the platform for 10 s. All mice were trained four times per day with an interval of more than 15 min for consecutive 5 days. The mice entered the water from four quadrants in turn, and randomly selected a point equidistant from the pool wall as the starting point in each quadrant. If the mice found the platform, the time they spent was recorded (defined as “escape latency”), which was used to assess their spatial learning ability. On the 6th day, the hidden platform was removed. The mice were set in the quadrant I and allowed to swim freely for 60 s for the probe test. The latency (escape latency) of the first arrival of the original platform position, the time required to swim across the original platform position, and the time animals stayed on the target quadrant within 60 s were recorded. After each test, the mice were dried. Behavior parameters were recorded and analyzed by behavioral analysis software (Any-Maze, United States).

The novel object recognition test (NORT) and object location test (OLT) are often used to evaluate spatial memory and discrimination. NORT was composed of an acquisition and recall period. The experimental apparatus was a square open field (50 cm in length, 50 cm in width and 50 cm in height) made of black polyvinyl chloride. Mice were acclimatized to the experimental room for at least 16 h and then placed in an open field for 10 min, which is 1 h before NORT. During the acquisition period, two identical green rectangular blocks were placed, respectively, at two corners of the same side, then each mouse was set in the square open field and allowed to explore freely for 10 min. The recall period was carried out 4 h later, with one object in each pair replaced by a novel object (a wooden red cone). Each mouse was placed in the open field for 10 min and the time spent exploring familiar and novel objects was recorded. The OLT was also composed of an acquisition and recall period. The treatment of the acquisition period was the same as NORT. During the recall period, each mouse was also placed into the open field for 10 min; however, the difference between NORT and OLT was that two original objects were placed diagonally in the OLT. The investigation time for novel object (%) = [time exploring the novel object/(time exploring the novel object + time to exploring the familiar object)] × 100%. Behavior parameters were recorded and analyzed using software (Any-Maze, United States) (Wan et al., 2021).

In the contextual fear condition (CFC), mice were trained and tested in a conditioning chamber, as previously reported (Yang et al., 2017). The experiment included a training and test period. The training period is composed of placing the mice in the chamber and delivering a footshock (2 s duration, 0.8 mA) without signal after 148 s of free exploration, and the mice were sent back to their home cage after free exploration for 30 s. During the test period, mice were placed in the same fear conditioning room (the contextual cue) for 180 s after 24 h. The frozen level (%) during the training period = [the freezing time of 148 s before the foot shock]/148 s × 100%. The frozen level (%) during the testing period = [the freezing time of 180 s during the testing period]/180 s × 100%. Freezing (defined as no movement at all, except breathing) was recorded and analyzed by the system software (Med Associates, Inc., United States).

### Injection of viral vectors

Mice were anesthetized with 2% isoflurane administered in 100% oxygen (4 l/min) for 1–2 min, maintained through a nose cone to optimize animal suffering. After the mice were deeply anesthetized, they were placed on the stereoscopic positioning instrument. The eyes were protected with aureomycin eye cream and the scalp was cut open. The fascia on the skull surface was removed by smearing with 3% hydrogen peroxide. The Bregma point and the Lambda point were used to adjust the mouse head to the horizontal position. The virus AAV-FBXO22-shRNA or AAV-FBXO22-scramble (SCM) was injected into bilateral hippocampus at a rate of 300 nl/min. Following the surgical procedure, the mice were immediately put on a warming pad (37°C). Bupivacaine (3 mg/kg) was injected subcutaneously into the incision site to reduce pain after surgery.

### Western blotting

In order to find the change of FBXO22 at early stage, western blot analysis was carried out at the end of propofol treatment (10, 25, 50, 100, and 200 mg/kg) once a day for 7 days, or 100 mg/kg once a day for 7 days in the FBXO22-shRNA rescue experiment, in an additional cohort of animals and not in the same animals underwent behavioral tests. Mice were decapitated after being deeply anesthetized with isoflurane. The hippocampus was isolated and homogenized and western blotting was conducted according to our previous study (Lu et al., 2017). Membranes were blocked with 5% BSA for 1 h at room temperature and then incubated overnight with 1:1000 of primary antibodies at least 12 h in blocking buffer at 4°C. FBXO22 antibody (Abcam Cat# ab230395, United States, Rabbit polyclonal), NMDAR1 antibody (Abcam Cat# ab16895, United States, Mouse monoclonal), PSD-95 antibody (1:000, CST Cat# 3450S, United States, Rabbit polyclonal), and β-actin antibody (1:5,000, Sigma-Aldrich Cat# A5316, United States) were utilized as the primary antibodies. After washing the blots with Tris-buffered saline with Tween 20 (1Tris-buffered saline containing 0.1% Tween 20), the membranes were incubated with peroxidase-labeled secondary antibodies (1,10,000, Sigma-Aldrich). The Quantity One image analysis program (Bio-Rad, United States) was used to analyze the signal intensity. β-actin was used to standardize protein amounts. Protein levels from the treatment group were expressed as a percentage in the control group.

### Primary cell culture and treatment

Dispersed hippocampal neurons were prepared from C57BL/6 as previously described (Chen et al., 2010). The hippocampal cultures of C57BL/6 mice were obtained from P0–P1 pups. Hippocampus was isolated and dissociated by 10% (v/v) trypsin (Life Technologies). After digestion for 30 min at 37°C, tissues were titrated using a 1 mL fire-polished pipette. For assessment of synapse morphology, hippocampal cultures were transfected with GFP-FBXO22-SCM and GFP-FBXO22-shRNA plasmid at 7 days *in vitro* and propofol (10 uM) was added in the dish at 14 days *in vitro* using the calcium phosphate method and fixed (4% formaldehyde, 4% sucrose in PBS, pH 7.4) at 21 days *in vitro*. A Z-stack of optical section was captured using a × 100 objective with a confocal microscope (Leica SP8). Quantitative analysis for dendritic spines was performed using the NeuronStudio software package, version 0.9.92 (Rodriguez et al., 2006).

### CA1 slice preparation

Mice injected with AAV-FBXO22-shRNA-GFP or AAV-FBXO22-shRNA-Scramble-GFP in CA1. 3 weeks later, the mice were treated with 100 mg/kg propofol once a day for 7 days, then they were anesthetized with pentobarbital (100 mg/kg, i.p.) and perfused transcardially with ice-cold oxygenated (95% O_2_/5% CO_2_) NMDG ACSF solution (Ting et al., 2018). After perfusion, the mice were decapitated, and the brain was immediately transferred to the same solution. The coronal plane of brain tissue was sectioned at 300 μm in the same buffer using a vibratome (VT1200 S, Leica). The brain slices containing hippocampal CA1 were cultured in oxygenated NMDG ACSF for 10–15 min at 32°C, then transferred to normal oxygenated ACSF (2.5 mM KCl, 126 mM NaCl, 2 mM MgSO_4_·7H_2_O, 1.25 mM NaH_2_PO_4_, 10 mM glucose, 2 mM CaCl_2_, 26 mM NaHCO_3_) at room temperature for 1 h. All chemicals were purchased from Sigma-Aldrich (St. Louis, MO, United States).

### *In vitro* electrophysiological recordings

The slices were shifted to the recording chamber which was superfused with aCSF at 28°C at 3 mL/min. The recorded neurons were identified by differential interference contrast optics (DIC; Olympus BX61WI) equipped with an GFP filter using infrared video microscopy and differential interference contrast optics fitted with a 40 × water-immersion objective. The recording pipettes (3–4 MΩ) were pulled with a micropipette puller (P2000, Sutter Instrument; United States). For whole-cell recording, the pipettes were filled with aCSF solution containing 18 mM NaCl, 133 mM potassium gluconate, 0.6 mM EGTA, 10 mM HEPES, 0.3 mM Na3·GTP and 2 mM Mg·ATP (pH 7.2, 280 mOsm). Under a voltage clamp mode, neurons were held at −70 mV to record spontaneous excitatory postsynaptic currents (sEPSC) for 5 min. Recordings with Rs >30 MΩ were excluded from the statistical analysis. A Multiclamp 700B amplifier and signals were low-pass filtered at 3 kHz and digitized at 10 kHz (DigiData 1,550, Molecular Devices) were used to acquire all recordings. Clampfit 10 software (Molecular Devices) and the Mini Analysis Program (Synaptosoft) were used to analyze the physiological data.

### Statistical analysis

For the behavioral studies, the number of mice in each group was 10. The number of samples was three in each group for western blotting, morphology of the spine, and *in vitro* electrophysiological recordings. The two-sided Student’s *t* test was used for two sample mean comparison with 5% type 1 error. The sample size of 10 mice in each group provided us with more than 80% statistical power to detect such a difference (Lu et al., 2017). Moreover, a sample size of three mice in each group provided the same statistical power for western blotting. A sample size of 13 neurons in each group provided the same statistical power for morphology of the spine (13 neurons from three mice), and *in vitro* electrophysiological recordings (13 neurons from three mice).

Assessment of the normality (D’Agostino and Pearson omnibus normality test) of the data was performed, *p* > 0.1 was considered to satisfy normality distribution. Statistical significance was determined by Two-way ANOVA followed by Sidak’s *post hoc* test for multiple comparisons when studying the effect of two factors, or by One-way ANONA followed by Sidak’s *post hoc* test when studying the effect of one factor. The study used a two-tailed hypothesis, and statistically significant *p*-values were less than 0.05. We evaluated all study data using Prism 6 software (GraphPad, United States).

## Results

### Long-term exposure to propofol impaired learning and memory

**Figure 2:**
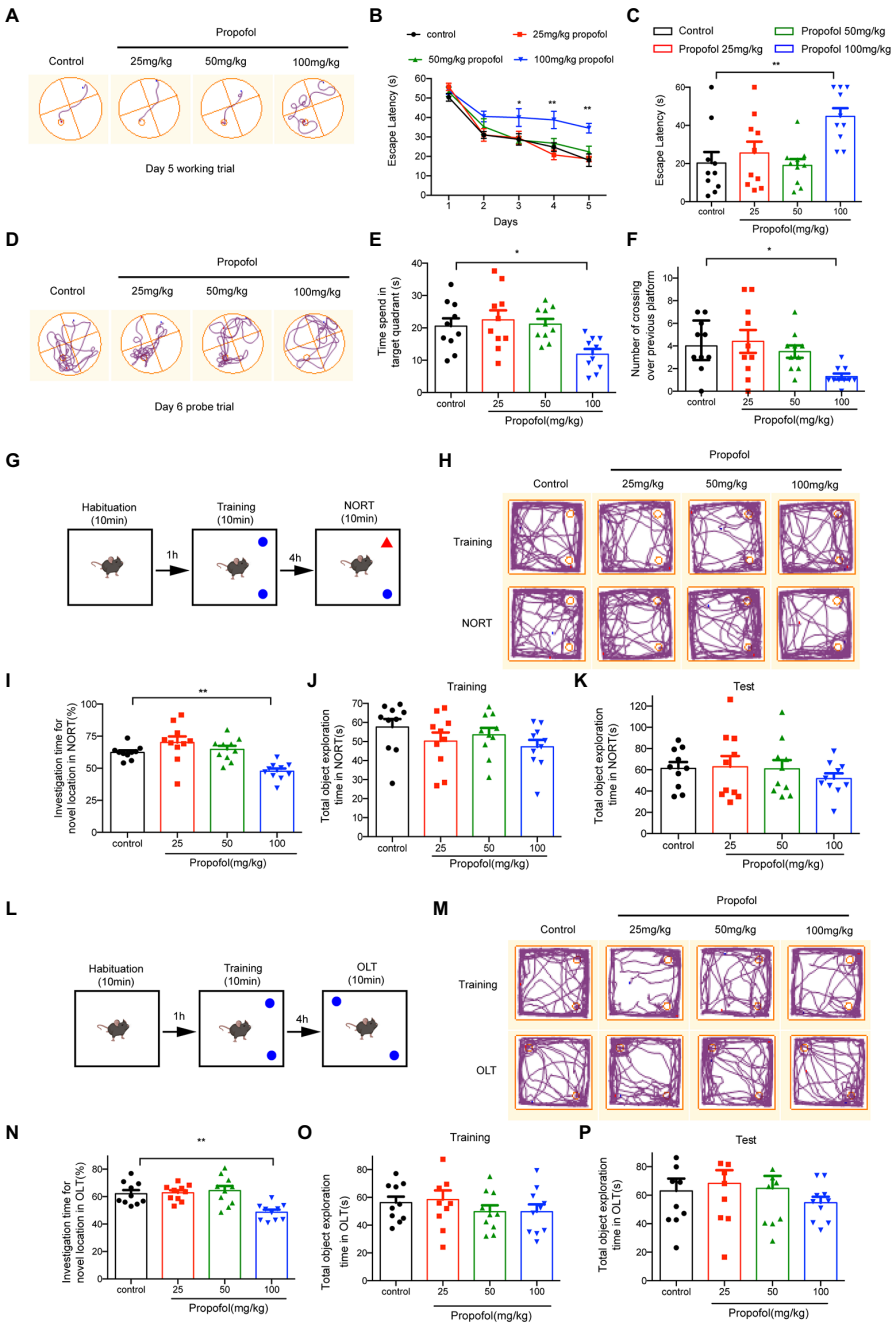
Effects of different doses of propofol (25, 50, and 100 mg/kg) anesthesia on spatial learning and memory in mice. **(A)** The typical trace of mice on the 5th training day. **(B)** The latency of finding the hidden platform from day 1 to day 5 during the training period. **(C)** The escape latency in the memory retrieval tests on the 6th day. **(D)** The typical paths of mice in the memory retrieval tests on the 6th day. **(E)** The time spent in the target quadrant on the test day. **(F)** The number of crossing over the previous platform on the test day. **(G,L)** The schematic diagram and representative paths traveled in NORT and OLT. **(H,M)** The typical paths of mice in training and test of NORT and OLT. **(I,N)** The investigation time for novel location in NORT and OLT. **(J,K)** The total object exploration time in NORT when training and testing. **(O,P)** The total object exploration time in OLT when training and testing. Data are presented as medians with interquartile range in panel F and are expressed as means ± SEM in other panels, and *n* = 10 mice per group; *p < 0 0.05, **p < 0 0.01, compared to the control group.

Morris water maze was used to examine propofol-impaired spatial learning and memory. As illustrated in Figures 2A–F, three different concentrations of propofol (25, 50, and 100 mg/kg) were administered intraperitoneally in this study. The latency of four groups to find the hidden platform gradually decreased with the progress of training (Figure 2B). From the third day, the hidden platform latency was prolonged in the propofol (100 mg/kg) group, while there were no significant differences between the 25 and 50 mg/kg propofol groups, compared to control group. (Two-way ANOVA followed by Sidak’s *post hoc* test for multiple comparisons, *F* = 15.960, *p* < 0.01, *n* = 10 mice/ group, Figures 2A,B). On day 6, in the memory retrieval test, the escape latency of the 100 mg/kg propofol group was significantly longer than that of the control group (One-way ANOVA followed by Sidak’s *post hoc* test for multiple comparisons, *F* = 6.593, *p* = 0.0011, =10 mice/ group, Figure 2C). Additionally, the time spent in the target quadrant and the number of crossings over the previous platform position in the 100 mg/kg propofol group were significantly lower than in the control group, while there were no significant differences between the 25 mg/kg and 50 mg/kg propofol groups and the control group (One-way ANOVA followed by Sidak’s *post hoc* test for multiple comparisons, *F* = 4.792, *p* = 0.0066 for the time spent in the target quadrant; *F* = 4.110, *p* = 0.0132 for the number of crossings over the previous platform position, *n* = 10 mice/ group, Figures 2D–F). MWM experiments demonstrated that long-term propofol anesthesia (100 mg/kg) significantly impaired spatial learning and memory in adult mice. NORT (Figures 2G,H) and OLT (Figures 2L,M) were used to assess the effects of long-term exposure to propofol on cognitive function in mice. No significant differences were observed in the total object exploration time of NORT and OLT between the four groups during the training and testing period (One-way ANOVA, *p* > 0.05, Figures 2J,K,O,P). However, the percentage of investigation time of new subjects in the NORT of adult mice given 100 mg/kg propofol for 7 consecutive days was lower compared to the control group (One-way ANOVA followed by Sidak’s *post hoc* test for multiple comparisons, *F* = 9.657, *p* < 0.0001, *n* = 10 mice/ group, Figure 2I). Similarly, the percentage of investigation time for a new location in OLT was also markedly reduced in the 100 mg/kg propofol group (One-way ANOVA followed by Sidak’s *post hoc* test for multiple comparisons, *F* = 8.604, *p* = 0.0002, Figure 2N). However, in NORT and OLT, there was no significant difference in the percentage of time to explore novel objects or novel locations between the 25 and 50 mg/kg propofol groups and the control groups. All these data suggest that the i.p. injection of propofol with 100 mg/kg for seven consecutive days could impair the spatial learning and memory ability in adult mice.

### Long-term exposure to propofol regulated the expression of FBXO22, PSD-95, and glutamate receptors

**Figure 3:**
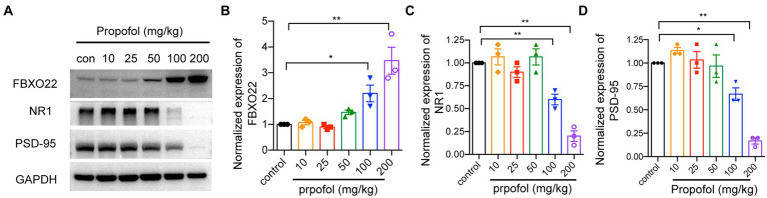
Western blot analysis of FBXO22, NMDAR1 and PSD95 protein level in hippocampus treated with different dose of propofol (10, 25, 50, 100, and 200 mg/kg). The protein level of FBXO22, NMDAR1 and PSD95 was determined by western blot **(A)** and the bands intensity corresponding to the target protein was analyzed **(B–D)**. Representative blots are shown. Data are expressed as means ± SEM, and *n* = 3 mice per group; *p < 0.05, **p < 0.01, compared to the control group.

To study the molecular mechanism underlying repeated propofol treatments-induced cognitive dysfunction, FBXO22, PSD-95, and NMDAR1 expression was detected by western blotting. As shown in Figures 3A–D, compared to intralipid-injected mice, injection of 100 and 200 mg/kg i.p. propofol increased FBXO22 and reduced the level of NMDAR1 and PSD-95 proteins (One-way ANOVA followed by Dunnett’s *post hoc* test for multiple comparisons, FBXO22: *F* = 14.990, *p* < 0.0001; NMDAR1: *F* = 27.800, *p* < 0.0001; PSD-95: *F* = 26.900, *p* < 0.0001, *n* = 3mice/ group). These data indicate that propofol (100 and 200 mg/kg) upregulated the expression of FBXO22 and suppressed the expression of PSD-95 and NMDAR1.

### Downregulation of FBXO22 restored the decreased glutamatergic synaptic transmission induced by propofol

**Figure 4:**
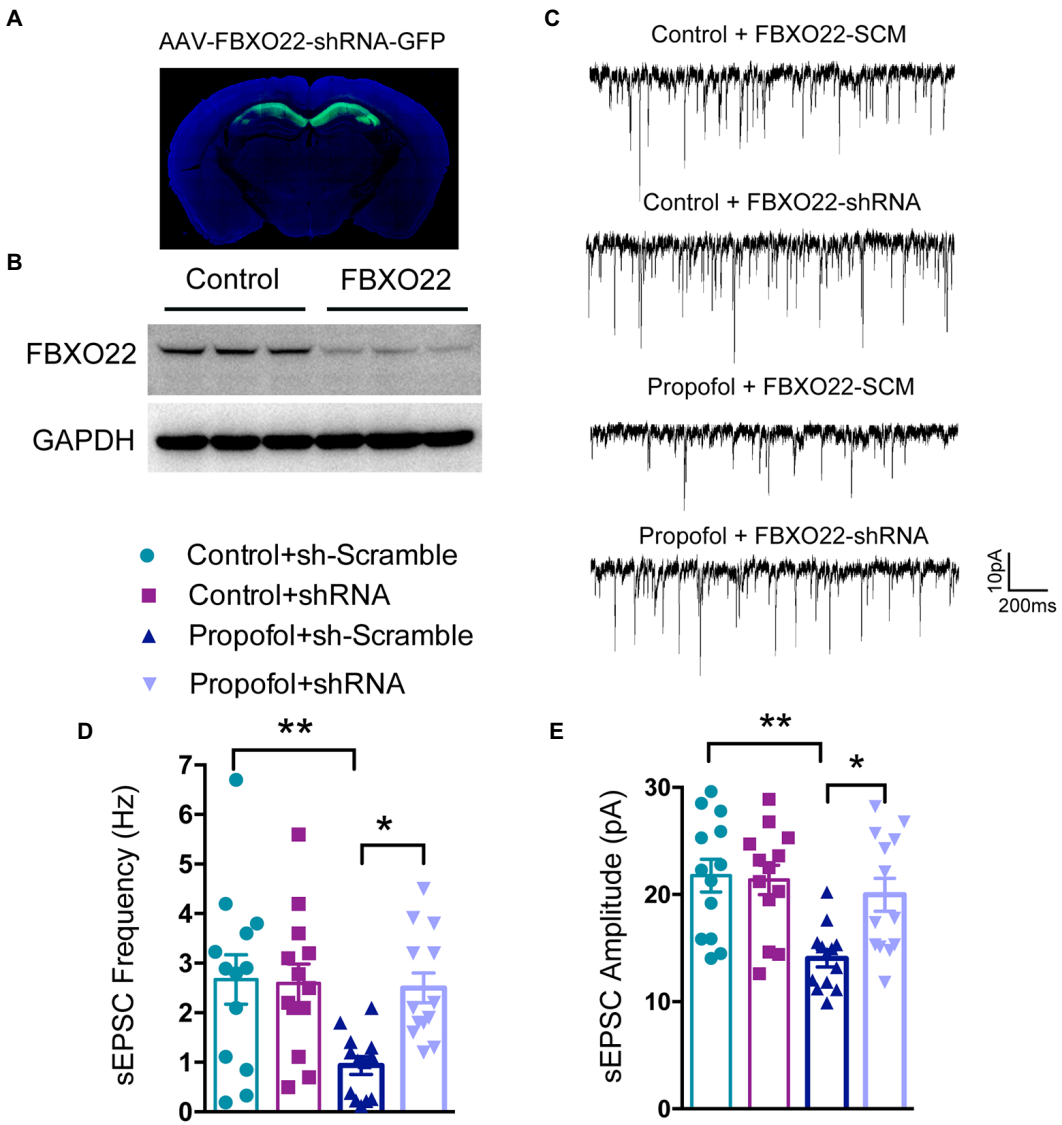
Downregulation of FBXO22 restored the defects of glutamatergic synaptic transmission induced by propofol. **(A)** Representative image of the mice’s brain slice injected with AAV-FBXO22-shRNA-GFP. **(B)** The FBXO22 protein level of mice injected with AAV-FBXO22-Scramble-GFP (control) and AAV-FBXO22-shRNA-GFP (FBXO22). **(C)** Representative sEPSC recordings for the control or propofol (100 mg/kg)treated, AAV-FBXO22-SCM-injected and AAV-FBXO22-shRNA-injected mice. **(D,E)** The quantitative analysis of mEPSC frequency and amplitude. Data are expressed as means ± SEM, and *n* = 13 neurons from three mice per group; *p < 0.05, **p < 0.01.

We next asked whether FBXO22 was necessary for regulating propofol-induced attenuation of excitatory synaptic transmission. To verify this mechanism, we measured sEPSCs in hippocampal CA1 acute brain slices obtained from mice injected with AAV-FBXO22-shRNA or AAV-FBXO22-SCM. The slices were subjected to voltage-clamp recordings *in vitro*. Compared to recordings from AAV-FBXO22-SCM-propofol-treated mice, whole-cell recordings from CA1 neurons from AAV-FBXO22-shRNA-propofol-treated mice showed a significant increase in sEPSC amplitudes and frequency (Two-way ANOVA followed by Sidak’s *post hoc* test for multiple comparisons, sEPSC frequency: *F* = 5.114, *p* = 0.0283; sEPSC amplitudes: *F* = 5.567，*p* = 0.0224, *n* = 13 neuron/ group, Figures 4A–E). These results suggest that the major change that occurs after FBXO22 knockdown is an increase in glutamatergic synaptic transmission, manifested as an increase in the number/function of postsynaptic receptors.

### Downregulation of FBXO22 restored decreased spine density in propofol-induced primary neurons of the hippocampus

**Figure 5:**
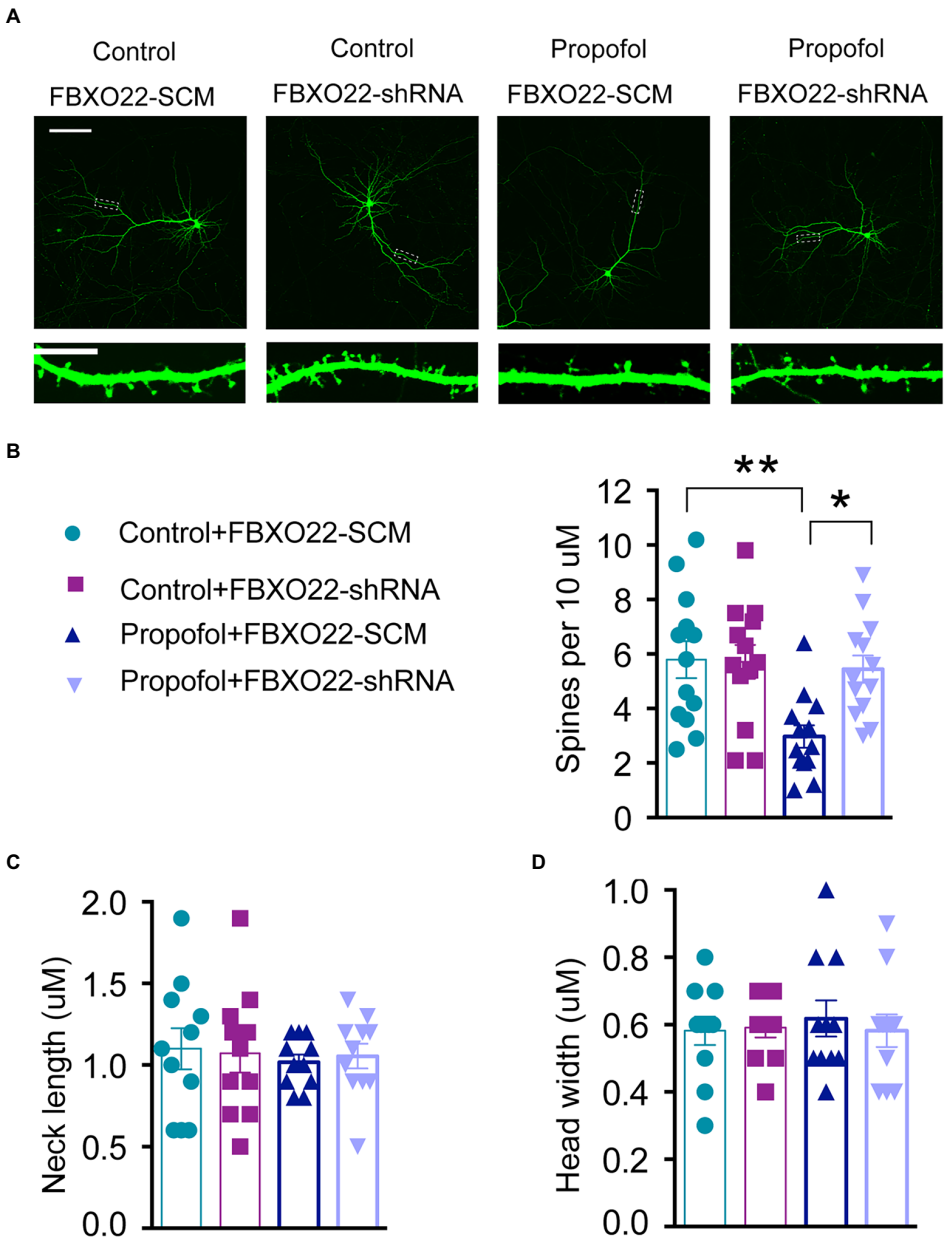
Downregulation of FBXO22 restored the defects of spine density in hippocampal primary neurons induced by propofol. **(A)** Representative images of the dendrites of primary hippocampal neurons transfected with either FBXO22-SCM or FBXO22-shRNA. Scale bars: upper panels, 25 μm; lower panels, 10 μm. **(B–D)** The summary of spine density, neck length, and head width. Data are expressed as means ± SEM, and *n* = 13 neuron from three mice per group; *p < 0.05, **p < 0.01.

Existing evidence indicates that the loss of dendritic spine density leads to decreased excitatory synapses (Sweet et al., 2009; Lo and Lai, 2020); thus, we examined whether FBXO22 expression leads to changes of dendritic spines in primary hippocampal cells. Consistent with the reduction in propofol-induced excitatory synaptic transmission, propofol treatment significantly decreased the dendritic spine density in neurons, which was reversed by knocking down FBXO22 expression with FBXO22-shRNA (Two-way ANOVA followed by Sidak’s *post hoc* test for multiple comparisons, *F* = 5.181, *p* = 0.0273, *n* = 13 neuron/ group, Figures 5A,B). Therefore, the downregulation of FBXO22 *in vitro* resulted in a significant increase in spine density when repeated treatment with propofol, while the morphology of spines (neck length and head width) showed no significant changes (Figures 5C,D).

### Downregulation of FBXO22 attenuated propofol-induced hippocampal-dependent cognitive dysfunction

**Figure 6:**
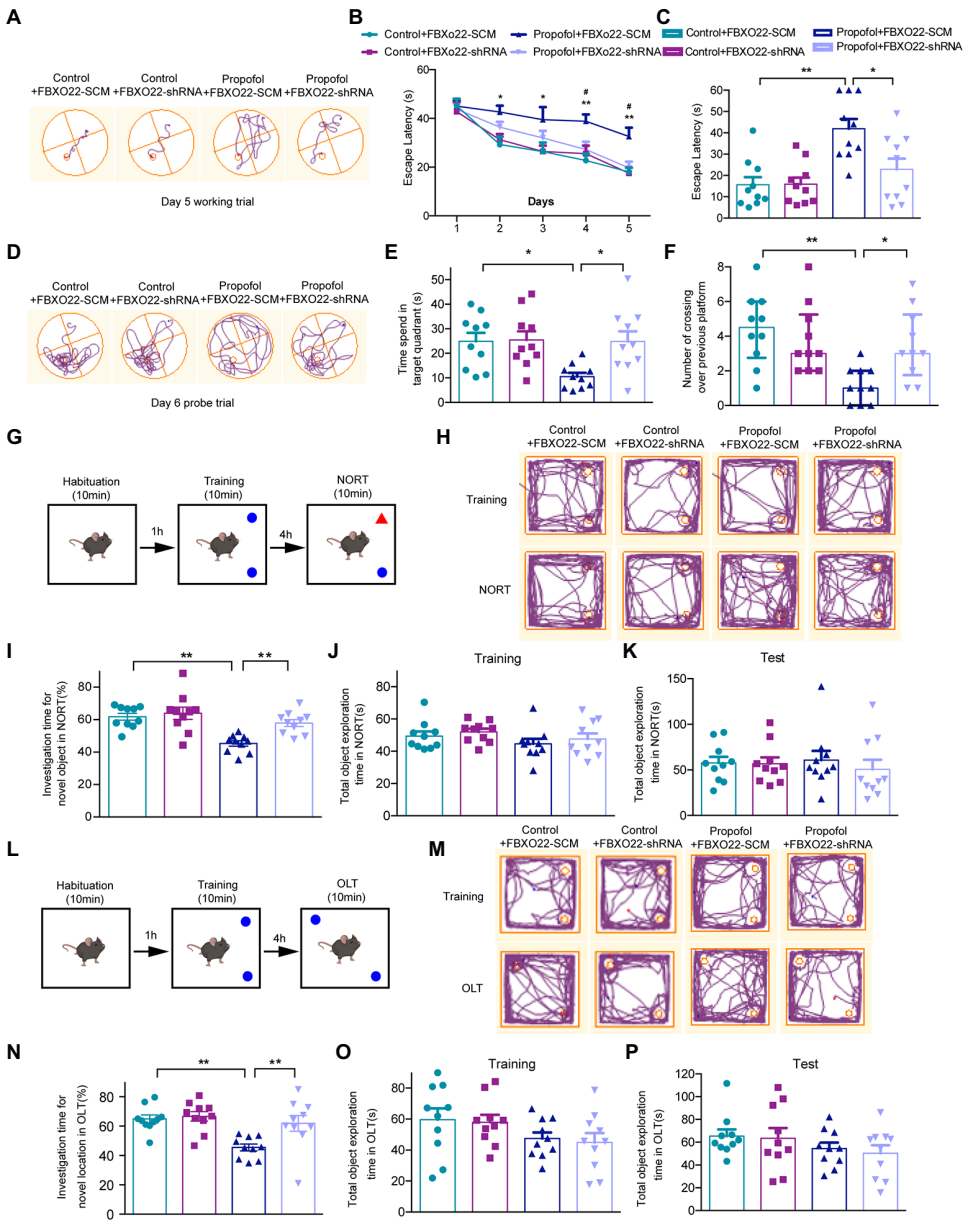
Downregulation of FBXO22 expression partially restored learning and memory ability induced by propofol (100 mg/kg). **(A)** The typical paths of mice on the 5th training day. **(B)** The latency of finding the hidden platform from day 1 to day 5 during the training period (Control+ FBXO22-SCM vs. Propofol+ FBXO22-SCM: *p < 0.05, **p < 0.01; Propofol+ FBXO22-SCM vs. Propofol+ FBXO22-shRNA: #*p* < 0.05). **(C)** The escape latency in the memory retrieval tests on the 6th day. **(D)** The typical paths of mice in the memorial retrieval test on the test day. **(E)** The time spent in the target quadrant in the memory retrieval tests. **(F)** The number of crossing over the previous platform position on the test day. **(G,L)** The schematic diagram and representative paths traveled in NORT and OLT. **(H,M)** The typical paths of mice in training and test of NORT and OLT. **(I,N)** The investigation time for novel location in NORT and OLT. **(J,K)** The total object exploration time in NORT when training and testing. **(O,P)** The total object exploration time in OLT when training and testing. Data are presented as medians with interquartile range in panel **F** and are expressed as means ± SEM in other panels, and *n* = 10 mice/ group. *p < 0.05, **p < 0.01.

To examine whether propofol-induced cognitive dysfunction was modulated by FBXO22, AAV-FBXO22-shRNA or AAV-FBXO22-SCM (scramble) were injected into the CA1 hippocampal region of mice. The latency of four groups of mice to find the hidden platform decreased daily with the progress of training (Figures 6A–C). From the second day of testing, the latency of finding the hidden platform in group propofol-FBXO22-SCM was longer than in the control-FBXO22-SCM group. Furthermore, on days 4 and 5 of testing, the latency of finding the hidden platform in group propofol-FBXO22-SCM was longer than in the propofol-FBXO22-shRNA group (Two-way ANOVA followed by Sidak’s *post hoc* test for multiple comparisons, *F* = 29.760, *p* < 0.0001, *n* = 10 mice/ group, Figures 6A,B). In the memory retrieval test on day 6, the escape latency of mice in the propofol+FBXO22-SCM group was longer than that of the Control+FBXO22-SCM group. However, the escape latency of mice in the propofol+FBXO22-shRNA group was shorter than that of the propofol+FBXO22-SCM group (Two-way ANOVA followed by Sidak’s *post hoc* test for multiple comparisons, *F* = 5.463, *p* = 0.0251, *n* = 10 mice/ group, Figures 6C,D). Furthermore, compared with the control-FBXO22-SCM group, mice in the propofol-FBXO22-SCM group remained within the target quadrant time and the number of crossings over the previous platform position within 60 s were significantly reduced. However, the time spent in the target quadrant (Two-way ANOVA followed by Sidak’s *post hoc* test for multiple comparisons, *F* = 4.299, *p* = 0.0454, *n* = 10 mice/ group, Figure 6E) and the number of crossings over the previous platform position within 60 s (Two-way ANOVA followed by Sidak’s *post hoc* test for multiple comparisons, *F* = 12.520, *p* = 0.0011, *n* = 10 mice/ group, Figure 6F) in group propofol-FBXO22-shRNA were more than those in group propofol-FBXO22-SCM.

No obvious differences were observed in the total object exploration time of NORT and OLT among four groups during the training and testing period (Figures 6J,K,O,P). However, in both NORT (Figures 6G,H) and OLT (Figures 6L,M), the percentage of investigation time of a new subject and a new location in the propofol-FBXO22-shRNA group was obviously higher than in the propofol-FBXO22-SCM group (Two-way ANOVA followed by Sidak’s *post hoc* test for multiple comparisons, NORT: *F* = 4.141, *p* = 0.0493; OLT: *F* = 4.132, *p* = 0.0495, *n* = 10 mice/ group, Figures 6I,N).

**Figure 7:**
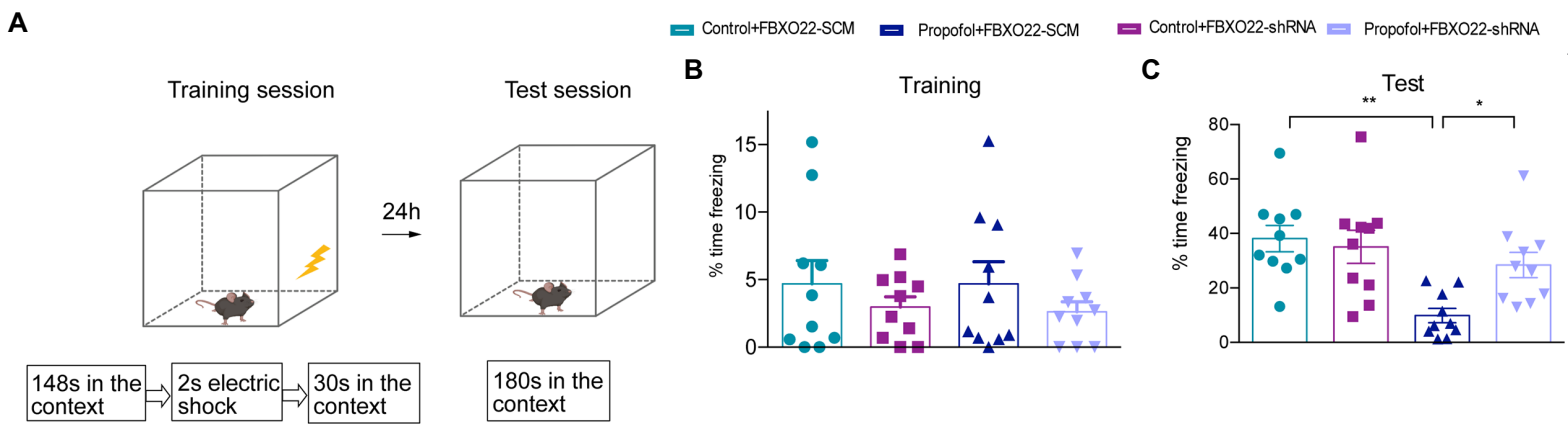
Downregulation of FBXO22 expression partially restored contextual memory induced by propofol (100 mg/kg). **(A)** The diagrammatic sketch of fear conditioning and context discrimination. **(B)** The baseline levels of fear behavior (freezing time)among four groups. **(C)** The freezing time during testing. Data are expressed as means ± SEM, and *n* = 10 mice per group; *p < 0.05, **p < 0.01.

The baseline levels of fear behavior (freezing time) among four groups did not show any difference in the training session (Figures 7A,B), and four groups all showed an increase in the freezing time in this context during the test session (Figure 7C). Additionally, the propofol-FBXO22-SCM group showed less freezing time than the control-FBXO22-SCM group, while the freezing time of the propofol-FBXO22-shRNA group was longer than that of the propofol-FBXO22-SCM group (Two-way ANOVA followed by Sidak’s *post hoc* test for multiple comparisons, *F* = 5.260, *p* = 0.0278, *n* = 10 mice/ group, Figure 7C). Taken together, these results suggest that long-term propofol anesthesia impairs hippocampal-dependent spatial learning and memory ability which is greatly improved with the knocking-down of FBXO22 expression.

## Discussion

Propofol is the most widely used intravenous general anesthetic and finds application in patients undergoing surgical procedures and invasive examinations (Emik et al., 2016). Although propofol has many desirable properties as a general anesthetic, there is growing evidence from animal studies suggesting that it may induce cognitive impairment, especially in younger and elder animals, while adult subjects are thought to be less vulnerable to this side effect (Le Freche et al., 2012; Ji et al., 2014; Lu et al., 2017). However, recent studies have found that prolonged isoflurane anesthesia (for up to 24 h) influences object recognition in mice of all ages, which is associated with increased synaptic turnover during anesthesia and synaptic loss during the postanesthetic period (Wenzel et al., 2021). This discovery updates our understanding and redirects our focus towards understanding changes in cognitive impairment associated with propofol treatment in adulthood.

In addition to age, both the anesthetic dose and the exposure time are also believed to have a great impact on cognition. A single exposure to anesthesia in childhood will not cause cognitive impairment, but a child exposed to anesthesia on more than three occasions may result in cognitive alterations (Wan et al., 2021). Therefore, the effects of different doses of propofol on cognitive function were studied after administering anesthesia up to 7 times in the present study. We found that intraperitoneal injection of 100 mg/kg propofol for 7 consecutive days could influence spatial learning ability in adult mice, and simulates the outcome of repeated clinical application of propofol. Considering that propofol is increasingly used in surgical procedures and invasive examinations, it is important to determine the safest doses of propofol for adult patients who require repeated general anesthesia in the clinical setting. Although our animal study cannot completely imitate the clinical impact of multiple anesthetics, our data suggest that adults who receive multiple consecutive exposures to propofol could have a higher risk of cognitive impairment. Our findings raise the warning that clinicians must pay attention to the dose of propofol administered to adults, especially during prolonged continuous treatment, and should be aware of possible cognitive impairment.

The mechanism of cognitive impairment induced by repeated administration of propofol remains unclear, making it difficult to preventively eliminate any detrimental effects that may emerge subsequently to the anesthetic process. Some studies support the concept that hippocampal synaptic plasticity disturbance (Uchimoto et al., 2014) plays an important role in cognitive impairment. Therefore, we examined the properties of primary neurons and hippocampal slices after propofol treatment in adult mice, including changes in the synaptic protein profile, dendritic spine density, and synaptic transmission.

We observed that repeated administration of propofol (above 100 mg/kg) to adults had an opposite regulatory effect on NMDAR1, PSD95, and FBXO22. These results indicate that increased FBXO22 expression was associated with repeated propofol treatment, while the levels of markers of synaptic plasticity decreased. Because most hippocampal neurons are excitatory neurons, it is necessary to record spontaneous excitatory postsynaptic currents (sEPSCs) to explore the effects of FBXO22 on the electrophysiology of neurons. A change in sEPSC frequency often predicts alterations in presynaptic release, while a change in the sEPSC amplitude often indicates alterations in postsynaptic receptor number or function. Our study showed that repeated treatment of propofol decreased both the frequency and amplitude of sEPSC, while knockdown of FBXO22 could rescue these phenomena. Furthermore, dendritic spines are dynamic structures in the brain (Dunaevsky et al., 1999; Matus, 2000), and their morphology and density have been implicated in long-term memory and cognition. Spine dynamics are also abnormal in several mental disorders (Penzes et al., 2011). Our study showed that propofol treatment significantly decreased dendritic spine density in neurons, while spine morphology (neck length and head width) did not show significant changes. These effects were reversed by knocking down the expression of FBXO22. Downregulation of FBXO22 may improve the cognitive deficits caused by propofol, indicating that the FBXO22-mediated protein degradation process also participates in memory formation.

It is worth noting that knockdown of FBXO22 expression did not significantly change the sEPSC frequency or amplitude, the dendritic spine density, the spine morphology, or cognitive function, but on repeated treatment with propofol, the knockdown of FBXO22 expression rescued the changes in all elements of the above profile. Therefore, the activity of FBXO22 was induced by repeated treatment of propofol. The focus of future research should be to investigate whether FBXO22 plays a role in other cognitive related diseases.

Numerous studies have described the role of protein degradation in activity-dependent synaptic plasticity (Cline, 2003; Cajigas et al., 2010). Ehlers et al., showed that chronic activation or inhibition of cultured hippocampal neurons leads to dynamic changes in the protein composition of postsynaptic density (PSD) (Stangier et al., 2003). Furthermore, inhibition of proteasome activity reverses many of these changes in synaptic structure. Furthermore, synaptic stimulation may lead to a redistribution of proteasomes from the dendritic axis to the spine, and the redistributed proteasomes become more active (Bingol and Schuman, 2006). Consistently, our study demonstrated that FBXO22 expression was upregulated, while that of PSD-95 was downregulated. The present study provides evidence indicating that protein degradation plays a pivotal role in the regulation of memory in the mammalian brain.

Limitations of the study should also be noted. First, propofol was administered before training, so the MWM findings suggest a direct association with hippocampus-dependent learning impairment, revealing the impact of propofol on the encoding of new information. Future studies could focus on memory consolidation or retrieval. Repeated administration of propofol between the training and test phases could reveal the potential effects of propofol on consolidation and recall once memory has been established. Second, due to the characteristics of E3 ligase, identifying the upstream and downstream components of the FBXO22 signaling pathway is very important to expand our understanding of cognitive function, which will be the research focus of our future studies. Third, we focused on the morphological and behavioral changes after repeated administration of propofol. Future studies should primarily focus on whether morphological and behavioral changes are reversible after the withdrawal of propofol.

In summary, the impact on cognitive impairment resulting from multiple administration of propofol in adult mice was evaluated in this study for the first time. Intraperitoneal injection of propofol (100 mg/kg) for 7 consecutive days impaired cognitive function, altered synaptic protein profiles, dendritic spine density, as well as synaptic transmission and increased expression of FBXO22 protein. This study proposes FBXO22 as a potential new target for the treatment of cognitive impairment.

## Data availability statement

The raw data supporting the conclusions of this article will be made available by the authors, without undue reservation.

## Ethics statement

The animal study was reviewed and approved by Laboratory Animal Ethics Committee of Xuzhou Medical University (L20210113006).

## Author contributions

XY, CC, and DQ performed behavioral experiments, analyzed data, and wrote the manuscript. YF and YZ conducted the western blot and analyzed data. YL and NW performed the *in-vitro* electrophysiology and analyzed data. HW examined the spine density in hippocampal primary neurons. BY analyzed and interpreted data. QX, YW, and HL conceived and designed the study. XY, YW, and HL obtained the funding. All authors contributed to the article and approved the submitted version.

## Funding

This work was supported by National Natural Science Foundation of China (81771138 to HL, 82171191 to YW, and 82001451 to XY) and Natural Science Foundation of Jiangsu Province (BK20191464 to YW).

## Conflict of interest

The authors declare that the research was conducted in the absence of any commercial or financial relationships that could be construed as a potential conflict of interest.

## Publisher’s note

All claims expressed in this article are solely those of the authors and do not necessarily represent those of their affiliated organizations, or those of the publisher, the editors and the reviewers. Any product that may be evaluated in this article, or claim that may be made by its manufacturer, is not guaranteed or endorsed by the publisher.
