# Supplementary figures and images for: Aberrant expression of FBXO22 is associated with propofol-induced synaptic plasticity and cognitive dysfunction in adult mice
Source: Front Aging Neurosci. 2022 Nov 8;14:1028148. doi: 10.3389/fnagi.2022.1028148 (PMC9680529; doi:10.3389/fnagi.2022.1028148)

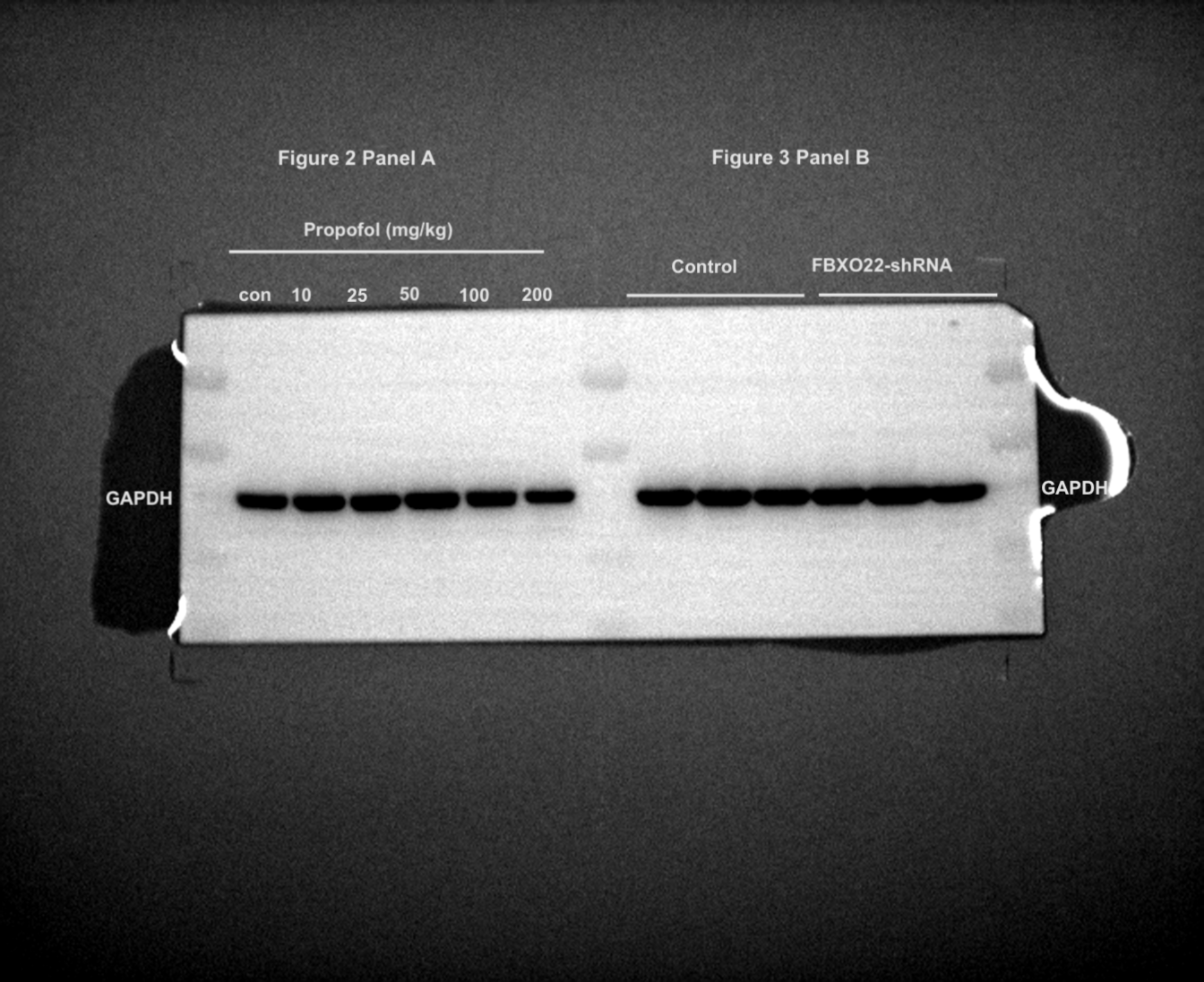

Supplement: Supplementary file 1 [file Data_Sheet_1.ZIP › original western blot images/GAPDH M.Tif]

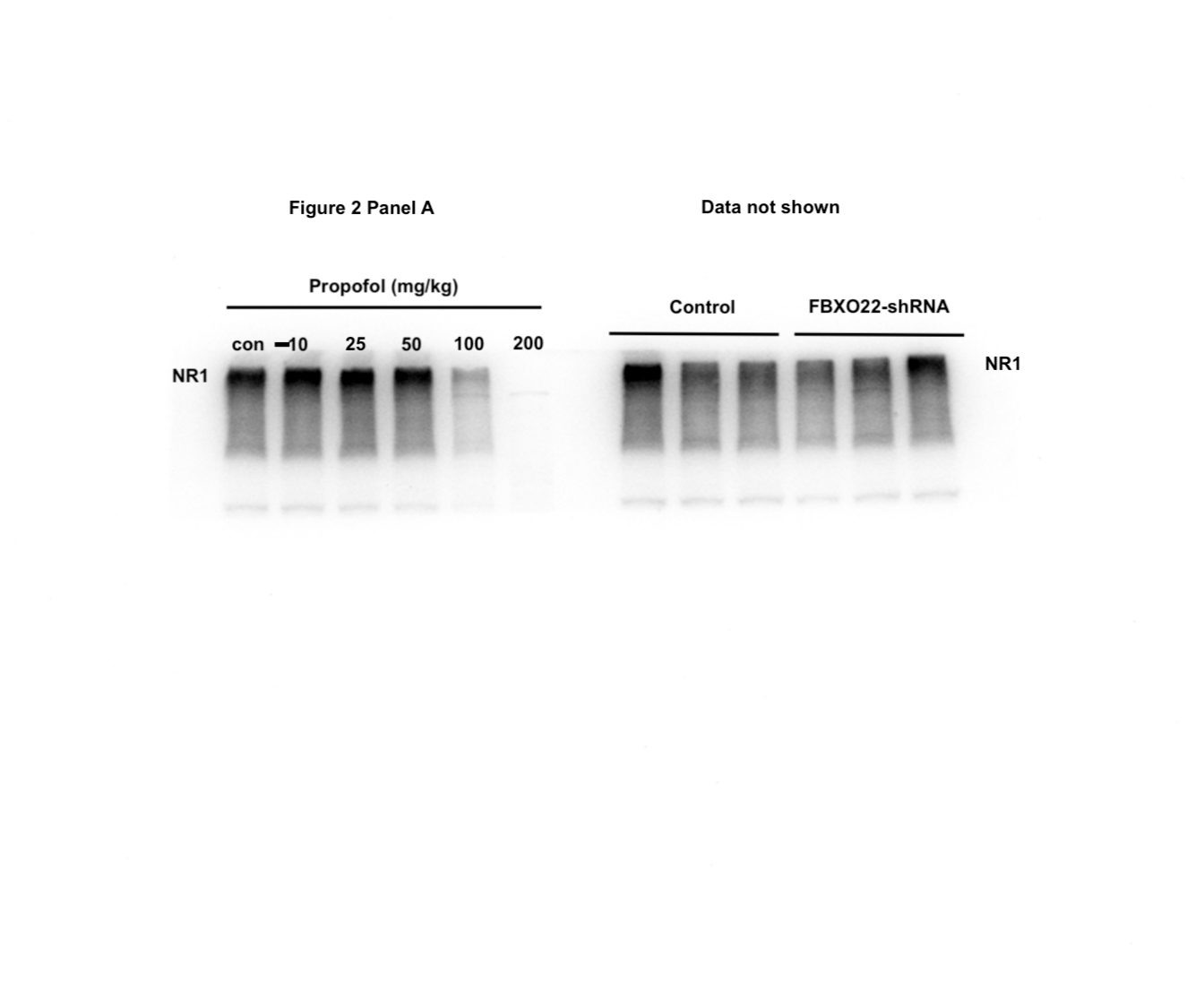

Supplement: Supplementary file 1 [file Data_Sheet_1.ZIP › original western blot images/NMDAR.Tif]

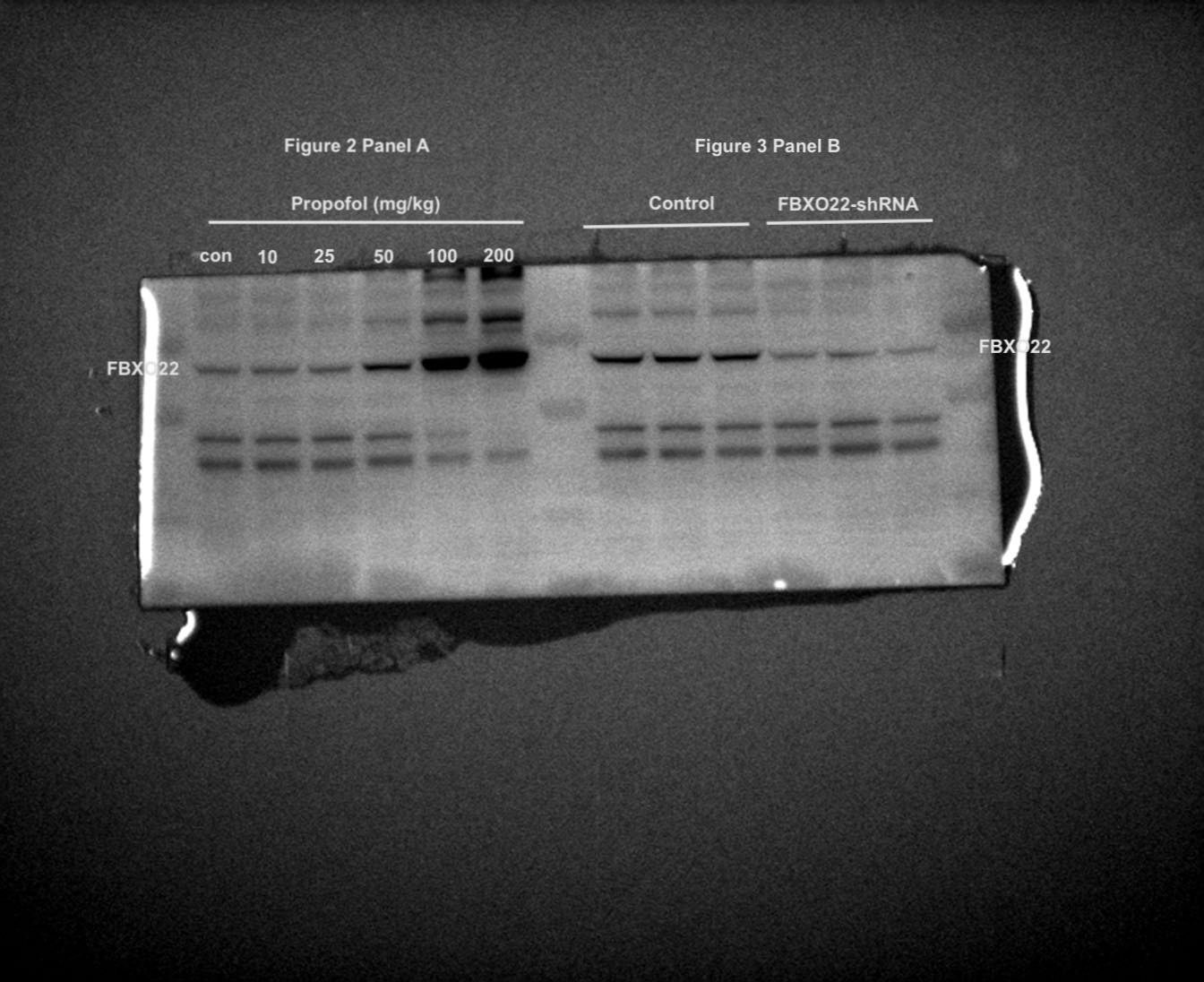

Supplement: Supplementary file 1 [file Data_Sheet_1.ZIP › original western blot images/FB.Tif]

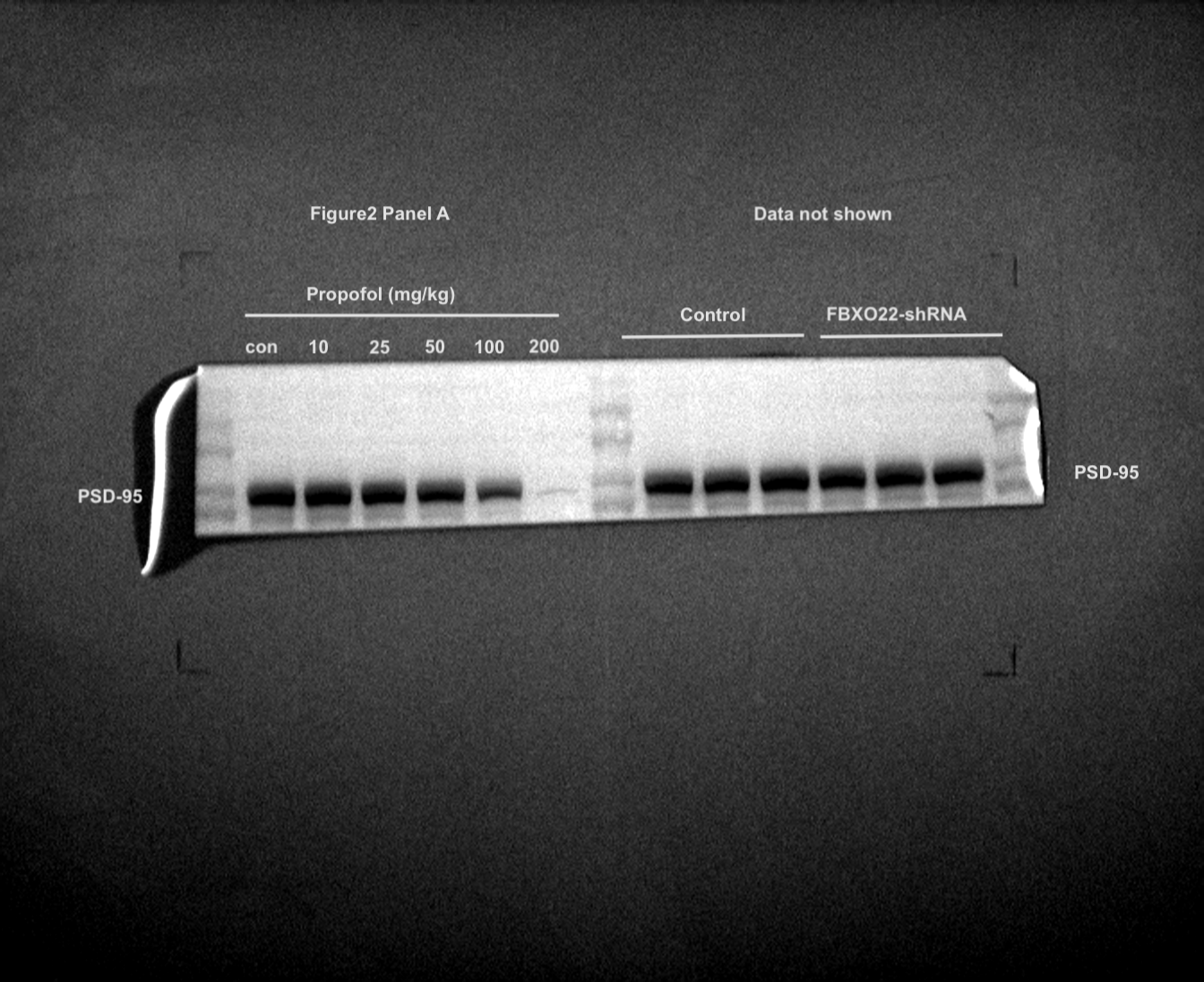

Supplement: Supplementary file 1 [file Data_Sheet_1.ZIP › original western blot images/PSD95 M.Tif]

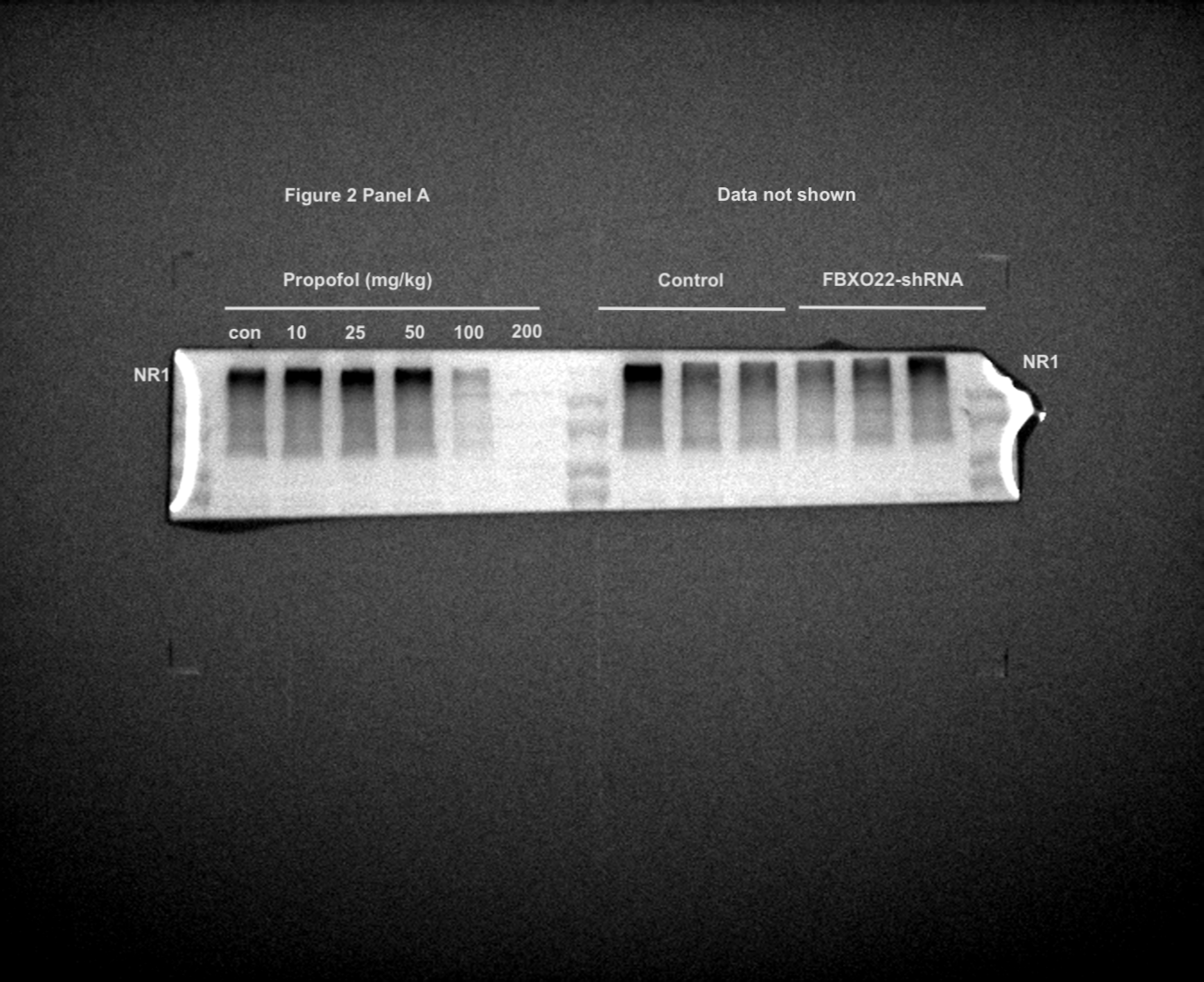

Supplement: Supplementary file 1 [file Data_Sheet_1.ZIP › original western blot images/NMDAR M.Tif]

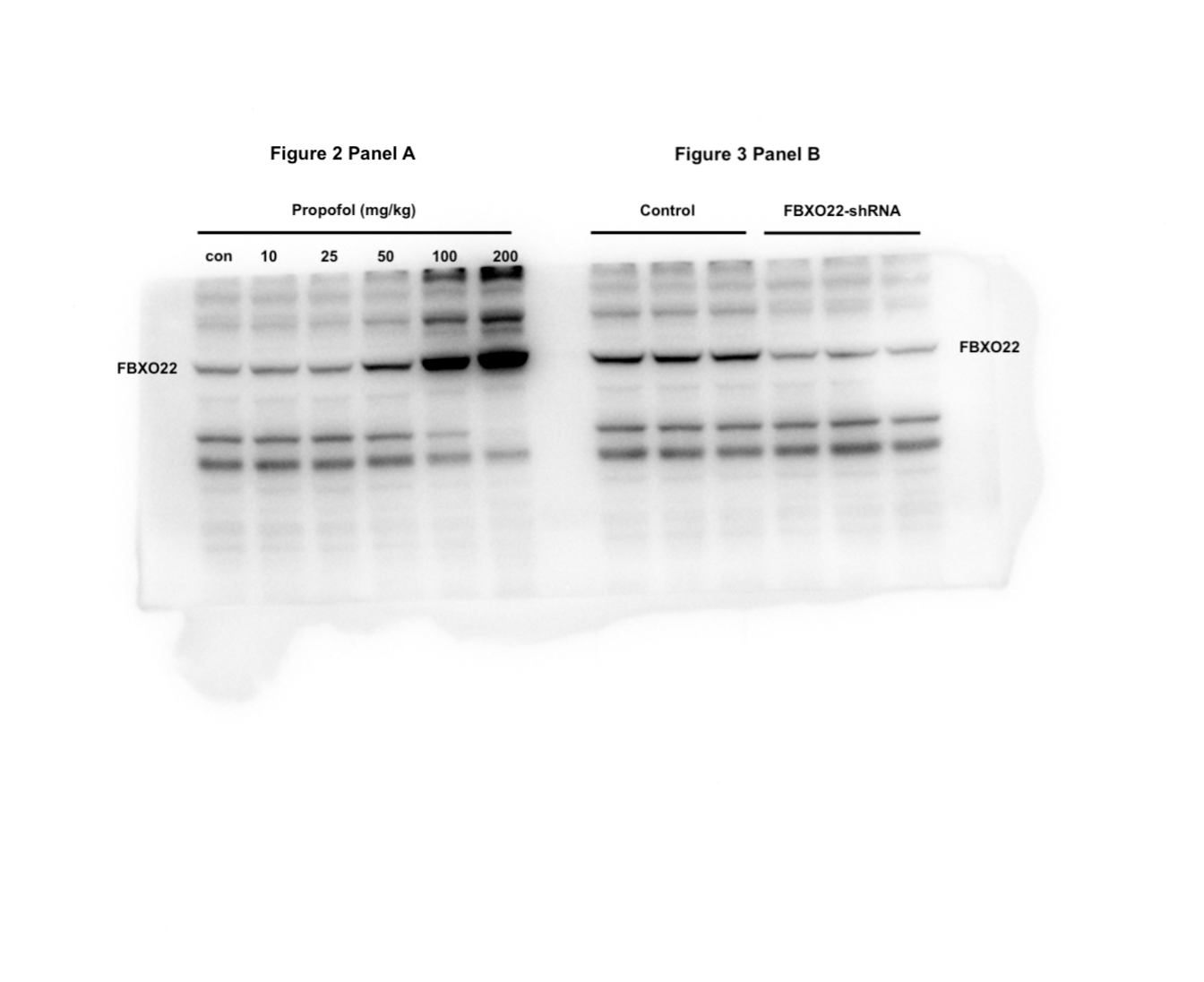

Supplement: Supplementary file 1 [file Data_Sheet_1.ZIP › original western blot images/FB M.Tif]

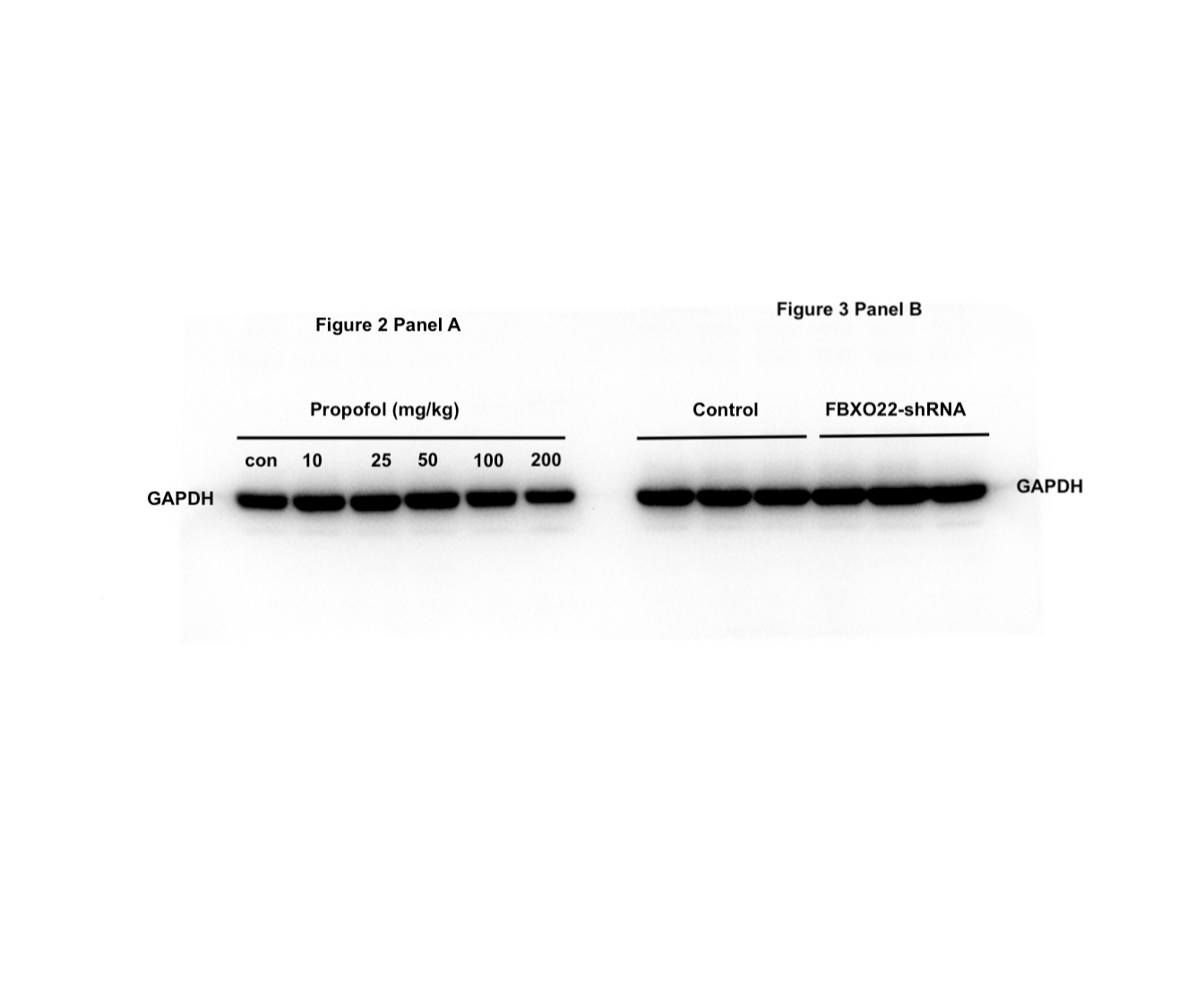

Supplement: Supplementary file 1 [file Data_Sheet_1.ZIP › original western blot images/GAPDH.Tif]

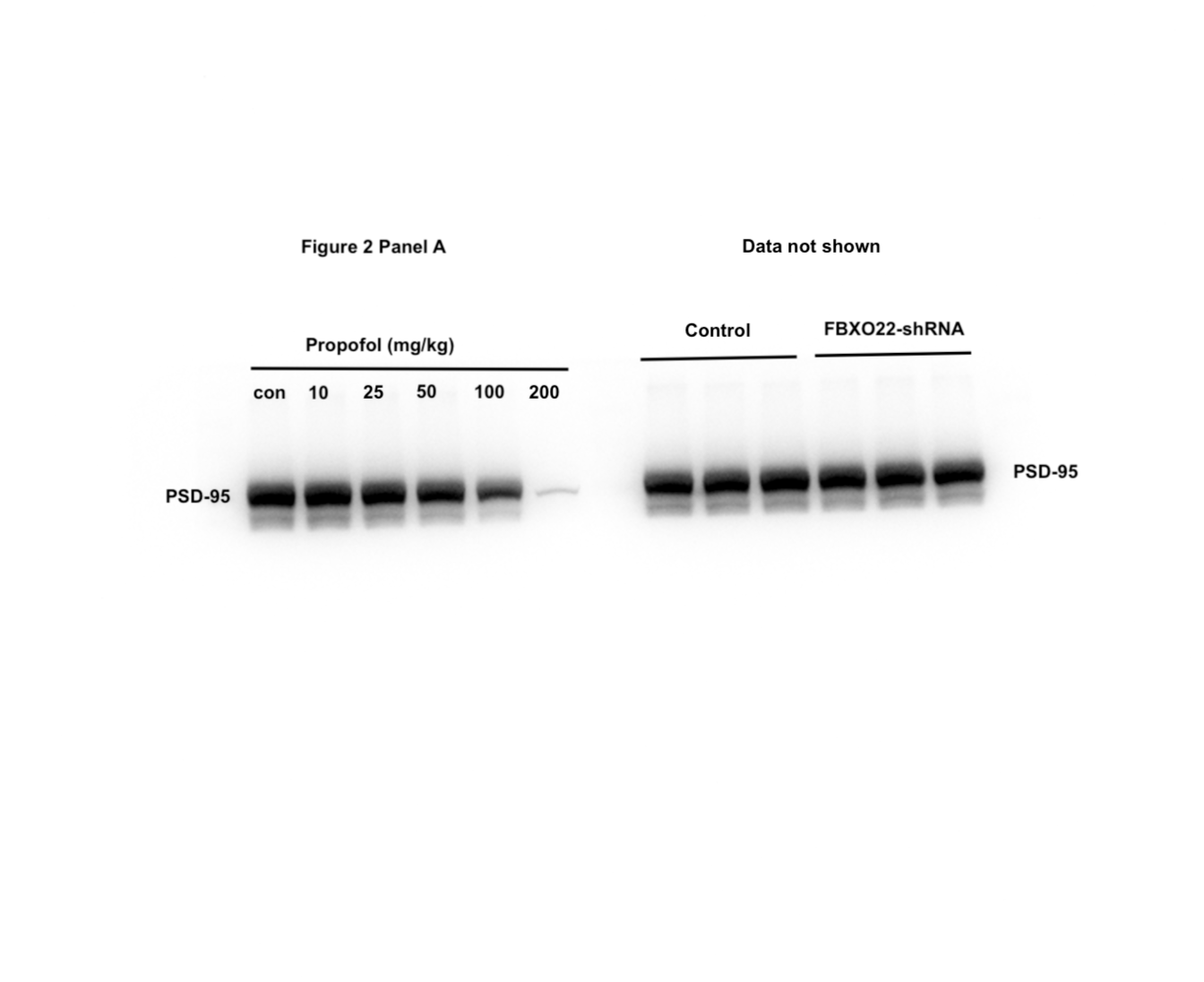

Supplement: Supplementary file 1 [file Data_Sheet_1.ZIP › original western blot images/PSD95.Tif]
